# Multiple Measures of Fixation on Social Content in Infancy: Evidence for a Single Social Cognitive Construct?

**DOI:** 10.1111/infa.12103

**Published:** 2015-09-22

**Authors:** Karri Gillespie‐Smith, James P. Boardman, Ian C. Murray, Jane E. Norman, Anne O'Hare, Sue Fletcher‐Watson

**Affiliations:** ^1^ University of the West of Scotland; ^2^ MRC Centre for Reproductive Health & Centre for Clinical Brain SciencesQueen's Medical Research InstituteUniversity of Edinburgh; ^3^ Department of Child Life and HealthUniversity of Edinburgh; ^4^ MRC Centre for Reproductive HealthQueen's Medical Research InstituteUniversity of Edinburgh; ^5^ The Salvesen Mindroom CentreUniversity of Edinburgh; ^6^ Patrick Wild CentreUniversity of Edinburgh

**Keywords:** social cognition, eye‐tracking, visual attention

## Abstract

The preference of infants to fixate on social information in a stimulus is well known. We examine how this preference manifests across a series of free‐viewing tasks using different stimulus types. Participants were thirty typically developing infants. We measured eye movements when viewing isolated faces, faces alongside objects in a grid, and faces naturally presented in photographed scenes. In each task, infants fixated social content for longer than nonsocial content. Social preference scores representing distribution of fixation to social versus general image content were highly correlated and thus combined into a single composite measure, which was independent of demographic and behavioral measures. We infer that multiple eye‐tracking tasks can be used to generate a composite measure of social preference in infancy. This approach may prove useful in the early characterization of developmental disabilities.

Infants preferentially direct their vision to faces from shortly after birth (Farroni, Csibra, Simion, & Johnson, 2002; Johnson, Dziurawiec Ellis, & Morton, 1991) showing a specific attentional focus on the eyes (Farroni et al., 2002). Indeed, from three months, infants are capable of distinguishing human from primate eyes and show a corresponding preference (Dupierrix et al., 2014). As infants get older, they also preferentially fixate faces in multiple‐object displays and animated scenes (Frank, Vul, & Johnson, 2009; Gliga, Elsabbagh, Andravizou, & Johnson, 2009). Infant fixation on social content is being developed to monitor development after preterm birth (De Schuymer, De Groote, Desoete, & Roeyers, 2012) and as a potential early marker of later autism spectrum disorder (ASD) diagnosis (Elsabbagh & Johnson, 2009; Jones & Klin, 2013). In this latter field, the role of fixation on the eye region as a key to social communication skill development is of particular interest (Senju & Johnson, 2009).

However, one interpretation of existing findings is that infants who later receive a diagnosis of ASD are more easily distracted by colorful objects in the background of a scene, or by specific stimulus properties such as audiovisual contingencies (Chawarska, Macari, & Shic, 2013; Klin, Lin, Gorrindo, Ramsay, & Jones, 2009). Thus, conflicting results from the literature on early signs of ASD may be partially explained by differences in stimulus and task design (Falck‐Ytter, Bolte, & Gredeback, 2013; Jones, Gliga, Bedford, Charman, & Johnson, 2014). It is essential to understand how stimulus design impacts on infant social fixation in typical development in order to provide a sound basis for explorations of atypical development.

We propose that combining data from different stimulus types may lead to a more robust measure of early infant preference for social content. This study combines established and novel eye‐tracking tasks to test the hypotheses that:

1. a preference to fixate on social information in infancy is consistent across stimulus types;
2. fixation on social information can be quantified by preference scores, which can be combined into a composite summary measure.

## Methods

### Participants

Typically developing infants were recruited from the community (mother and baby groups). Inclusion criteria were as follows: singleton birth at more than 36 completed weeks’ postmenstrual age, aged 6–12 months at time of assessment. Exclusion criteria were known chromosomal abnormalities and suspected or confirmed neurodevelopmental delay. Ethical approval was given by the University of Edinburgh, School of Education ethics committee, and written informed consent was obtained from parents or guardians.

### Procedure

Infants either stood or sat on their mother's laps approximately 50–60 cm from the monitor and watched a series of images, while their eye movements were recorded. Prior to data collection, an eye‐tracking calibration was performed using a five‐point system and inspected by the researcher. Infants viewed stimuli until they had either seen them all or became distracted/unsettled. Breaks were given when necessary.

Mothers completed a background questionnaire regarding: maternal education, ethnicity, socioeconomic status, and family history of neurodevelopmental conditions. In addition, they completed: a measure of infant temperament, the very short form of the Infant Behavior Questionnaire Revised (Gartstein & Rothbart, 2003) (IBQ‐R); a measure of parenting stress, the Parenting Daily Hassles Scale (Crnic & Greenberg, 1990), and the Edinburgh Postnatal Depression Scale (Cox, Holden, & Sagovsky, 1987). These measures were intended to explore independence of infant social attention preferences from temperamental and demographic characteristics.

### Tasks

**Figure 1:**
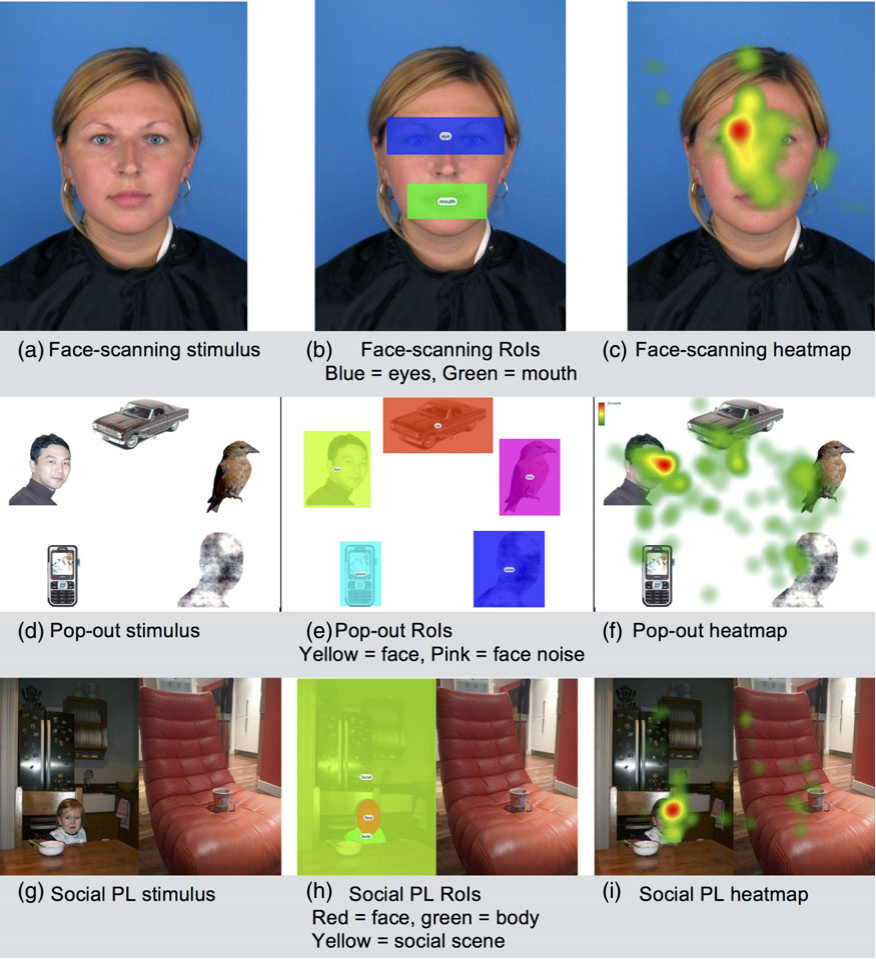
Sample stimuli, regions of interest and heatmaps for the face‐scanning task (a–c), the pop‐out task (d–f), and the social preferential looking task (g–i). This panel shows example stimuli for each task. We have written consent from adults, and from the parents of children, shown in these images.

The study employed three free‐viewing tasks, each using a different type of social stimulus, described below with examples shown in Figure 1. Each task was presented in blocks, referring to a short run of different stimuli from a single task. For example, a block of two face‐scanning stimuli, followed by a block of three pop‐out stimuli and so on. Between stimuli, attention grabbers were shown to maintain the infant's focus on the screen. These were moving cartoon images of toys on a black background, accompanied by nonsocial sound effects and displayed for 1 sec (between trials in a block) or 3 sec (between blocks). The three tasks presented were interleaved with others not reported here. Total eye‐tracking time was approximately 18 min presented in 3, 6‐min sequences.

### Social stimuli and tasks

#### Face scanning: free viewing of natural faces

Photographs of faces were selected from the 2D face database at the University of Stirling.^1^ The resulting stimuli depicted direct gazing male and female faces with neutral expressions, with an on‐screen size of 16 cm × 21.5 cm (see Figure 1). These stimuli (*N* = 6) were presented for 10 sec each.

#### Pop‐out: free viewing of isolated natural faces alongside other objects in a grid‐like display

Stimuli (*N* = 10) showed photographs of faces, animals, and objects against a white background, provided by the British Autism Study of Infant Siblings (BASIS) (Elsabbagh et al., 2013; Gliga et al., 2009). Nonsocial content included a car, mobile phone, and bird as well as a “face‐noise” image. This was a control image which had the same dimensions and low‐level visual properties as a face but was scrambled so that it was unrecognizable (see Figure 1). Stimuli were sized 28 cm × 21 cm on screen and were presented for 10 sec each.

#### Social preferential‐looking: free viewing of two photographs side‐by‐side, with and without social content

This task was adapted from an adult version (Fletcher‐Watson, Findlay, Leekam, & Benson, 2008; Fletcher‐Watson, Leekam, Benson, Frank, & Findlay, 2009) using a smaller set of images and featuring children instead of adults. The stimuli were 12 pairs of photographs of real‐world scenes (see Figure 1). Each pair contained a social scene (depicting one or two children) and a nonsocial scene (depicting no people). Stimuli were created for this study by taking photographs of everyday scenes both with and without children—thus, each photograph is partnered with a “control” photograph of the same location, but without people. When creating the stimuli used in this study, photographs were shuffled such that each social scene was paired with a nonsocial scene from a different setting. This process controls for stimulus complexity across the whole stimulus set. The final stimuli were sized 24 cm × 17 cm on screen and were presented for 5 sec each, slightly longer than the adult version of this task to account for potentially slower performance in infants.

### Apparatus

Eye movements were detected by a Tobii© X60 eye tracker. Tobii Studio (Falls Church, VA, USA) (Falls Church, VA, USA) 3.1.0 software was used to present stimuli and record the eye movements for analysis. The eye tracker was controlled by a Dell Optiplex 745. Images were presented on an HP Compaq LA1905wg monitor with screen size width 40.8 cm and height 25.0 cm and resolution 1440 × 900 pixels. The Tobii x60 system tracks both eyes to a rated accuracy of 0.3°, sampled at 60 Hz.

### Analysis methods

Stimuli were organized into regions of interest (RoIs) for subsequent analysis, using Tobii Studio definition tools. Eye‐tracking data comprised fixation durations on each RoI within a stimulus, and on the whole stimulus (i.e., all RoIs plus all areas not covered by an RoI). In addition, the time taken to first fixate each RoI was extracted. We excluded all first fixation times less than 100 ms, as these do not represent the result of voluntary, planned eye movements to a specific region (Liversedge & Findlay, 2000). In addition, individual trials on which total fixation duration on the whole stimulus was less than 500 ms were excluded, for the same reason. A small proportion of trials were excluded in this way (face scanning = 4%; pop‐out = 2%, social PL = 8%).

Normality was assessed using measures of skew and kurtosis and by visual inspection of histograms and Q‐Q plots. Where data did not meet the normality assumption, Wilcoxon signed‐rank tests were used to test for within‐group differences between conditions and we report medians and interquartile ranges. Otherwise analyses employed *t*‐tests to explore differences between conditions, and Pearson's correlations to test for relationships between tasks and with background variables. Where necessary, a Bonferroni correction for multiple comparisons was applied. Confidence intervals for correlations were derived using an online calculator (http://vassarstats.net/rho.html).

**Figure 2:**
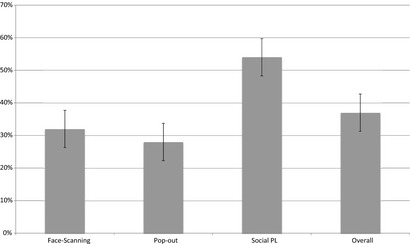
Percentage preference scores for social content across tasks (*N* = 30). (1) Face‐scanning social preference = Fixation Duration to Eyes/Total Fixation Duration. (2) Pop‐out social preference = Fixation Duration to Face/Total Fixation Duration. (3) Social PL social preference = Fixation Duration to Social Scene/Total Fixation Duration.

After analyzing each task's data, we created social preference scores by calculating the percentage of mean total time spent looking at the “most social” area of a scene (as defined below in the numerator of each listed calculation) versus mean total looking time for that scene type (see Figure 2). These represent a hierarchy of social interest from scenes containing people, to faces, to specific face regions. Calculations were as follows:

1. Face‐scanning social preference = Fixation Duration to Eyes/Total Fixation Duration
2. Pop‐out social preference = Fixation Duration to Face/Total Fixation Duration
3. Social PL social preference = Fixation Duration to Social Scene/Total Fixation Duration.

Social preference scores were related to demographic and behavioral variables of interest: gender, age at testing, birthweight, and parent‐reported infant temperament.

## Results

Thirty typically developing infants aged 6.1–12.3 months old were recruited. All infants were able to comply with task demands and be calibrated successfully. The majority of the infants completed all social tasks in full (*N* = 20), and the remainder viewed either 2 blocks per task (*N* = 9) or 1 block per task (*N* = 1). No mothers screened positive for postnatal depression, and all scored in the low range on parental stress. Data are summarized in Table 1.

**Table 1:** Background Characteristics of the Sample (*N* = 30)

		Ratio	Mean	*SD*
Infant Characteristics	Age (months)	n/a	8.3	1.7
	Gender (m:f)	15:15	n/a	n/a
	Birthweight (grams)	n/a	3601	398
	Breastfeeding ceased (before 6mo: after 6mo)	10:20	n/a	n/a
Maternal Characteristics	Age (years)	n/a	32.9	3.6
	Ethnicity (White British: Other)	28:2	n/a	n/a
	Highest educational qualification (Postgraduate^a^: Other)	12:18	n/a	n/a
	Most recent employment level (Professional: Other)	23:7	n/a	n/a
Parenting Daily Hassles	Frequency	n/a	34.2	7.3
	Intensity	n/a	29.5	7.7
Postnatal Depression	Total	n/a	2.97	2.6
Infant Behaviour Questionnaire	Surgency	n/a	4.83	0.72
	Negative Affect	n/a	3.34	0.88
	Effortful Control	n/a	5.18	0.68

*Note*.

a Masters, PhD, or professional qualification.

### Normality of data

Overall fixation durations and time to first fixate for whole stimuli were normally distributed. However, there was evidence of skew and kurtosis in the distributions of eye‐tracking fixation durations and time to first fixate on specific RoIs for all three tasks (Table 2).

**Table 2:** Eye‐Tracking Task Scores (*N* = 30; all Scores in Seconds)

Task	Measure	AOI	Median	IQR
Face‐scanning	Fixation durations	Eyes	1.35	0.54–3.02
		Mouth	0.07	0.00–0.32
		Whole display	5.71	4.41–7.01
	Time to first fixate	Eyes	1.74	0.88–3.29
		Mouth	3.51	1.90–5.22
Pop‐out	Fixation durations	Face	1.48	0.63–2.87
		Face‐Noise	0.44	0.05–0.63
		Bird	0.26	0.14–0.51
		Car	0.25	0.12–0.53
		Phone	0.12	0.06–0.27
		Whole display	5.96	4.58–7.14
	Time to first fixate	Face	2.18	1.43–2.93
		Face‐Noise	3.04	1.61–4.20
		Bird	3.91	2.37–5.08
		Car	3.16	2.36–4.63
		Phone	3.81	3.33–5.70
Social PL	Fixation durations	Social scene	1.43	1.05–2.20
		Nonsocial scene	0.70	0.50–0.89
		Faces	0.14	0.00–0.49
		Bodies	0.18	0.08–0.29
		Whole display	2.56	2.00–3.36
	Time to first fixate	Social scene	1.34	0.77–1.62
		Nonsocial scene	1.42	1.17–1.87
		Faces	1.95	1.29–2.23
		Bodies	1.66	1.03–2.49

### Face scanning

Wilcoxon signed‐rank tests showed that infants fixed on the eyes for significantly longer (*p* < .001) and more rapidly after image onset (*p* = .02) compared with the mouth. In this task, 87% (*N* = 26) of participants made their quickest fixations to the eyes or to the top half of the face. There were no significant correlations between age at testing or birthweight and eye‐tracking measures (see Table S1).

### Pop‐out task

Median fixation duration on the face was higher than for any other region of interest (see Table 3). A series of one‐sample Wilcoxon signed‐rank tests with Bonferroni correction (*p* = .05/4 = alpha value for significance of .0125) compared fixation duration for each area of interest with the known median fixation duration on the face. These demonstrated that each area of interest was fixated significantly less than the face (all *p* < .001). Likewise, Wilcoxon signed‐rank tests using the same adjusted alpha revealed that the bird, car, and phone (see Figure 1) produced times to first fixate that were on average slower than those to the face (all *p* < .001). However, there was no significant difference between time taken to fixate the face and the face noise (*p* = .061). In this task, 30% (*N* = 9) of participants made their quickest fixations to the face, and 30% (*N* = 9) made their quickest fixations to the nonface RoI. There were no correlations between age at testing or birthweight and eye‐tracking measures (see Table S2).

**Table 3:** Bivariate Correlations Between Fixation Durations to Social and Nonsocial Display Elements Across Eye‐Tracking Tasks

		Face scanning		Pop‐Out			Social PL		
		Eyes	Mouth	Face	Face‐noise	Car	Face	Social scene	Nonsocial scene
Eyes	Pearson Correlation	1	−.100	.667^a^	.546^a^	.281	.858^a^	.464^b^	−.188
	95% CI for *r*		−.440 to .269	.404 to .828	.232 to .757	−.088 to .582	.721 to .930	.125 to .706	−.513 to .184
	Sig. (2‐tailed)		.598	.000	.002	.132	.000	.010	.319
	*N*	30	30	30	30	30	30	30	30
Mouth	Pearson Correlation		1	.241	.146	.140	−.148	−.153	−.123
	95% CI for *r*			−.130 to .553	−.226 to .480	−.231 to .476	−.482 to .224	−.486 to .219	−.462 to .248
	Sig. (2‐tailed)			.200	.441	.460	.434	.421	.517
	*N*		30	30	30	30	30	30	30
Face	Pearson Correlation			1	.546^a^	.113	.562^a^	.266	−.178
	95% CI for *r*				.232 to .757	−.257 to .454	.253 to .766	−.104 to .571	−.505 to .194
	Sig. (2‐tailed)				.002	.554	.001	.156	.345
	*N*			30	30	30	30	30	30
Face‐noise	Pearson Correlation				1	−.067	.608^a^	.504^b^	−.071
	95% CI for *r*					−.417 to .300	.318 to .794	.176 to .731	−.420 to .296
	Sig. (2‐tailed)					.726	.000	.005	.709
	*N*				30	30	30	30	30
Car	Pearson Correlation					1	.115	−.105	.003
	95% CI for *r*						−.255 to .456	−.265 to .448	−.357 to .362
	Sig. (2‐tailed)						.546	.580	.988
	*N*					30	30	30	30
Face	Pearson Correlation						1	.618^a^	−.198
	95% CI for *r*							.332 to .800	−.521 to .174
	Sig. (2‐tailed)							.000	.293
	*N*						30	30	30
Social scene	Pearson Correlation							1	−.025
	95% CI for *r*								−.381 to .388
	Sig. (2‐tailed)								.895
	*N*							30	30
Nonsocial scene	Pearson Correlation								1
	95% CI for *r*								
	Sig. (2‐tailed)								
	*N*								30

*Notes*.

a Significant comparisons at *p* = .00185 (Bonferroni adjustment for 27 comparisons).

b Significant comparisons at *p* = .01 (unadjusted).

### Social PL

Wilcoxon signed‐rank tests revealed a significant difference in fixation duration on each scene (*p* < .001). However, there was no difference in mean time to first fixate each scene (*p* = .175). In this task, 66% of participants (*N* = 20) made their quickest fixations to the social scene. There were no correlations between age at testing or birth weight and eye‐tracking measures (see Table S3).

### Social preference variables

These variables were all normally distributed (Figure 2). Bivariate correlations showed that social preference scores were significantly correlated with each other for all three tasks (face scanning with pop‐out: *r *=* *.638, *p* < .001, 95% CI .361 to .811; face‐scanning with social PL: *r *=* *.620, *p* < .001, 95% CI .335 to .801; pop‐out with social PL: *r *=* *.497, *p* = .005, 95% CI .167 to .727). These significant relationships held when partial correlations were performed, firstly controlling for average fixation duration across all tasks and RoIs (i.e., a measure of general attentiveness to the screen; all *r *>* *.44 and all *p* < .017) and secondly controlling for age at testing (all *r *>* *.54 and all *p* < .003; see Table S4 for full details).

To investigate whether links between tasks were specific to social content, we also ran correlations of unadjusted fixation durations to both social and nonsocial regions of stimuli. Following a Bonferroni correction for multiple correlations (*p* = .05/27 = alpha level for significance of .00185), there were significant correlations across different eye‐tracking tasks in looking to faces embedded in social scenes, looking to faces in pop‐out stimuli, and looking to the eye region of faces (Table 3). In contrast, correlations between fixation durations on nonsocial (or less social—e.g., the mouth) regions were uniformly nonsignificant.

### Reliability of tasks

To assess whether this collection of tasks exhibited internal reliability, we performed a split‐half analysis. Taking stimuli in each task in order of presentation, we split these into two groups using an alternate selection procedure (i.e., odd numbered stimuli in group one, even numbered stimuli in group two). Pearson's correlations were significant for every RoI assessed (see Table 4), with the exception of fixation duration to the mouth in the face‐scanning task, indicating high levels of consistency within each task.

**Table 4:** Bivariate Correlations Between fixation Durations on Split‐half Data for Each Eye‐tracking Task and ROI Separately (Correlations Reported are for Each ROI Correlated With Itself for Odd and Even Numbered Stimuli)

	Face Scanning		Pop‐out			Social PL	
	Eyes	Mouth	Face	Face‐noise	Car	Social scene	Nonsocial scene
Pearson Correlation	.579^a^	.237	.646^a^	.377^a^	.415^a^	.762^a^	.419^a^
95% confidence interval	.277 to .777	−.134 to .550	.373 to .816	.020 to .649	.065 to .674	.554 to .880	.070 to .677
Sig. (two‐tailed)	.001	.224	.000	.040	.022	.000	.021
*N*	28^b^	28^b^	30	30	30	30	30

*Notes*.

a Significant comparisons at *p* = .05.

b Two infants did not view enough stimuli in one of the split‐half stimulus groups to be included in this calculation.

We attempted to replicate this analysis using data on the time taken to first fixate each RoI. This yielded significant correlations between each stimulus set for the Social PL task only (social scene *r *=* *.762, *p* < .001; nonsocial scene *r *=* *.419, *p* = .021). However, for the face‐scanning and pop‐out task, data available for the analyses were limited due to exclusion of cells where time to first fixate was less than 100 ms (see Analysis methods), and therefore, no significant relationships were found.

To further explore the internal consistency of each task, we re‐calculated all fixation duration and time to first fixate data on an image‐wise basis (i.e., mean scores for each stimulus, averaged across all participants). This process tests for images that elicit a pattern of eye movement responses that differ from other stimuli within the same task. Visual inspection of the data revealed normal distributions and no systematically outlying stimuli of concern. In the face‐scanning task, there was no difference between fixation durations and time to first fixate within each RoI when comparing female and male faces (t‐tests, all *p* > .15) and no evidence of outlying stimuli. One stimulus in the pop‐out task produced much longer fixation durations to the car than other stimuli within the task (average car fixation duration = 0.42 sec, fixation duration on car for stimulus 6 = 1.32 sec). Inspection of this image shows that the car here is bright red, which may have made it more engaging to infants than the same RoI in other stimuli. There were no outlying stimuli in the social PL task.

### Relationship between social cognition, infant demographics, and measures of infant behavior

A single, normally distributed social preference composite score was created by averaging social preference scores across the three eye‐tracking tasks (Figure 2). T‐tests between groups revealed no effect of age group (based on median split) nor infant gender on social preference composite. In addition, social preference score did not correlate with parent‐reported infant temperament (IBQ‐R surgency, *r *=* *.290, *p* = .127, 95% CI −.078 to .588; IBQ‐R negative affect, *r *=* *.093, *p* = .632, 95% CI −.276 to .438; IBQ‐R effortful control, *r *=* *.179, *p* = .353, 95% CI −.193 to .506), infant age at testing (*r *=* *.046, *p* = .809, 95% CI −.319 to .399), nor infant weight at birth (*r *=* *−.183, *p* = .342, 95% CI −.509 to .189).

## Discussion

This study demonstrates consistent evidence of rapid and extended fixation on social content relative to nonsocial content, across three free‐viewing tasks employing stimuli of differing visual layouts and social content. No such consistent relationship was found in fixation to nonsocial content in the same images. These stimuli could be characterized as demonstrating increasing ecological validity from isolated faces to images of people in natural scenes. However, we did not extend this process to include moving stimuli nor concurrent audio content. Our interpretation of the mouth as less socially informative than the eyes is partly dependent on the use of static images, as the mouth is particularly informative in contexts where language is being used (Lewkowicz & Hansen‐Tift, 2012). Thus, a different definition of socially informative regions might apply if using moving images.

Social preferences, measured by fixation on what we term the most socially informative areas of stimuli, relative to stimuli as a whole, were correlated between tasks despite the differences in stimulus type. These correlations facilitated creation of a single, combined social preference score reflecting fixation on social content across stimuli and tasks. This measure was independent of infant age, birthweight, and temperament. A social preference score arrived at in this manner may represent a more powerful and comprehensive measure of infant social ability than scores based on a single stimulus set.

### Influence of stimulus design on fixation

Our data indicate some subtle consequences of stimulus design on gaze behavior. The pop‐out stimuli present a face and also a face‐noise image, sharing the same low‐level visual properties of the face but scrambled to remove social meaning. Infants in this study were equally likely to look at either of these images first and showed no difference in the average time taken to do so. Likewise, the social preferential‐looking (social‐PL) task revealed no significant difference in time taken to first look at the social and nonsocial scenes. Thus, a preference for social information is constrained by the capacity of the visual system to identify social content from peripheral vision. The influences of *preference for* social content and *capacity to detect* social content may be important when investigating the role of eye‐tracking measures as biomarkers of later function.

### Limitations of the current study

This work is a preliminary contribution intended to enhance the way in which early preference for social information in infancy is measured and used to provide an estimation of later difficulty. Our findings are based on a small sample, and a small stimulus set presented for a brief time. In particular, the small number of stimuli presented for the face‐scanning and pop‐out tasks prevented accurate exploration of split‐half reliability of a time to first fixate measure. In the future, eye‐tracking measures should be validated against real‐world social cognitive tests such as parent–child interactions, and the discriminant validity of these measures when used with atypical populations must be determined. The infants we assessed were born to mothers with at least a college‐level qualification who reported low scores on measures used to evaluate maternal postnatal depression, maternal stress, and infant temperament. Further studies are required to define the limits of typical development of social cognition and to explore its development under nonoptimal conditions.

### Evidence for a single social cognition construct measured by fixation

Eye‐tracking provides a useful system for making inferences about cognition in infancy. In this case, a robust and independent preference for the most socially informative areas of a stimulus was apparent. There was no such pattern in looking to nonsocial content. We interpret these data as providing evidence for a single social cognitive construct which operates across tasks. Employing multiple measures like this may be useful for identifying infants at risk of later impairment.

## Author Contributions

All listed authors were involved in conception and design of the study and had final approval of the paper before submission. In addition, KGS recruited participants and collected data and SFW performed analysis and provided the first full draft of the manuscript.

## Supporting information


**Table S1.** Correlations between eye‐tracking variables from the face scanning task, with age at testing and birth weight.
**Table S2.** Correlations between eye‐tracking variables from the pop‐out task, with age at testing and birth weight.
**Table S3.** Correlations between eye‐tracking variables from the social PL task, with age at testing and birth weight.
**Table S4.** Partial correlations between social preference scores for each task controlling for (a) attentiveness and (b) age at testing.

## Note

1
http://pics.psych.stir.ac.uk
