# Supplementary material for: Multiple Measures of Fixation on Social Content in Infancy: Evidence for a Single Social Cognitive Construct?
Source: Infancy. 2015 Sep 22;21(2):241–57. doi: 10.1111/infa.12103 (PMC4762533; doi:10.1111/infa.12103)
Supplement: Supplementary file 1 — Table S1. Correlations between eye‐tracking variables from the face scanning task, with age at testing and birth weight. Table S2. Correlations between eye‐tracking variables from the pop‐out task, with age at testing and birth weight. Table S3. Correlations between eye‐tracking variables from the social PL task, with age at testing and birth weight. Table S4. Partial correlations between social preference scores for each task controlling for (a) attentiveness and (b) age at testing. [file INFA-21-241-s001.docx]

Supplementary Table 1: Correlations between eye-tracking variables from the face scanning task, with age at testing and birth weight

|  | | **Fixation duration** | | | **Time to first fixate** | |
| --- | --- | --- | --- | --- | --- | --- |
|  | | **eyes** | **mouth** | **All ROIs combined** | **Eyes** | **Mouth** |
| age in days at testing | Pearson Correlation | .072 | -.210 | -.046 | .090 | .181 |
|  | 95% CI for *r* | -.295 - .401 | -.523 - .171 | -.399 - .319 | -.285 - .441 | -.312 - .597 |
|  | Sig. (2-tailed) | .705 | .266 | .811 | .643 | .472 |
|  | N | 30 | 30 | 30 | 29^ | 18^ |
| weight at birth in grams* | Pearson Correlation | -.203 | -.255 | -.170 | -.085 | .180 |
|  | 95% CI for *r* | -.530 - .176 | -.568 - .123 | -.505 - .209 | -.443 - .297 | -.329 - .608 |
|  | Sig. (2-tailed) | .291 | .182 | .379 | .667 | .490 |
|  | N | 29 | 29 | 29 | 28^ | 17^ |

^ reduced sample sizes for time to first fixate data reflect the fact that any trials on which the infant was already looking at the ROI at the point of stimulus onset were excluded from analysis. These trials would produce a time to first fixate of zero seconds which would skew the mean downwards if left in the analysis.

* birth weight data were not available for one infant

Supplementary Table 2: Correlations between eye-tracking variables from the pop-out task, with age at testing and birth weight

|  | | **Fixation duration** | | | **Time to first fixate** | | |
| --- | --- | --- | --- | --- | --- | --- | --- |
|  | | **Car** | **Face** | **Noise** | **Car** | **Face** | **Noise** |
| age in days at testing | Pearson Correlation | -.208 | -.141 | .179 | -.105 | .329 | -.073 |
|  | 95% CI for *r* | -.528 - .164 | -.477 - .231 | -.193 - .506 | -.466 - .286 | -.042 - .620 | -.471 - .349 |
|  | Sig. (2-tailed) | .270 | .457 | .345 | .602 | .081 | .733 |
|  | N | 30 | 30 | 30 | 27^ | 29^ | 24^ |
| weight at birth in grams* | Pearson Correlation | -.212 | -.130 | -.026 | -.239 | .230 | .256 |
|  | 95% CI for *r* | -.536 - .167 | -.473 - .248 | -.388 - .343 | -.573 - .163 | -.156 - .555 | -.174 - .604 |
|  | Sig. (2-tailed) | .269 | .503 | .893 | .239 | .239 | .239 |
|  | N | 29 | 29 | 29 | 26^ | 28^ | 23^ |

NB: the Car was chosen to represent all non-social content on the slide. Similar results were apparent for other non-social ROIs (bird, phone).

^ reduced sample sizes for time to first fixate data reflect the fact that any trials on which the infant was already looking at the ROI at the point of stimulus onset were excluded from analysis. These trials would produce a time to first fixate of zero seconds which would skew the mean downwards if left in the analysis.

* birth weight data were not available for one infant

Supplementary Table 3: Correlations between eye-tracking variables from the social PL task, with age at testing and birth weight

|  | | **Fixation duration** | | **Time to first fixate** | |
| --- | --- | --- | --- | --- | --- |
|  | | **Non social scene** | **Social scene** | **Nonsocial scene** | **Social scene** |
| age in days at testing | Pearson Correlation | .094 | .290 | .206 | .098 |
|  | 95% CI for *r* | -.276 - .438 | -.078 - .588 | -.166 - .527 | -.271 - .442 |
|  | Sig. (2-tailed) | .620 | .120 | .276 | .608 |
|  | N | 30 | 30 | 30 | 30 |
| weight at birth in grams* | Pearson Correlation | .026 | .032 | .099 | .248 |
|  | 95% CI for *r* | -.343 - .388 | -.338 - .393 | -.277 - .449 | -.130 - .563 |
|  | Sig. (2-tailed) | .892 | .870 | .610 | .194 |
|  | N | 29 | 29 | 29 | 29 |

* birth weight data were not available for one infant

Supplementary Table 4: Partial correlations between social preference scores for each task controlling for a) attentiveness and b) age at testing

| **Variable 1** | | | Face Scanning social preference | Pop-out social preference | Social PL social preference |
| --- | --- | --- | --- | --- | --- |
| **Variable 2** | | | Pop-out social preference | Social PL social preference | Face Scanning social preference |
| **Control variable** | Average total fixation duration across tasks | Correlation | .607 | .441 | .593 |
|  |  | Significance (2-tailed) | .000 | .017 | .001 |
|  |  | df | 27 | 27 | 27 |
|  | Age at testing | Correlation | .653 | .535 | .620 |
|  |  | Significance (2-tailed) | .000 | .003 | .000 |
|  |  | df | 27 | 27 | 27 |
